# An efficient protocol for *Agrobacterium*-mediated transformation of the biofuel plant *Jatropha curcas* by optimizing kanamycin concentration and duration of delayed selection

**DOI:** 10.1007/s11816-015-0377-0

**Published:** 2015-11-06

**Authors:** Qiantang Fu, Chaoqiong Li, Mingyong Tang, Yan-Bin Tao, Bang-Zhen Pan, Lu Zhang, Longjian Niu, Huiying He, Xiulan Wang, Zeng-Fu Xu

**Affiliations:** Key Laboratory of Tropical Plant Resources and Sustainable Use, Xishuangbanna Tropical Botanical Garden, Chinese Academy of Sciences, Menglun, 666303 Yunnan China; College of Life Science and Agriculture, Zhoukou Normal University, Zhoukou, 466001 Henan China; University of Chinese Academy of Sciences, Beijing, 100049 China

**Keywords:** Biofuel, Cotyledon, Delayed selection, Physic nut, Transformation, Kanamycin concentration

## Abstract

*Jatropha curcas* is considered a potential biodiesel feedstock crop. Currently, the value of *J. curcas* is limited because its seed yield is generally low. Transgenic modification is a promising approach to improve the seed yield of *J. curcas.* Although *Agrobacterium*-mediated genetic transformation of *J. curcas* has been pursued for several years, the transformation efficiency remains unsatisfying. Therefore, a highly efficient and simple *Agrobacterium*-mediated genetic transformation method for *J. curcas* should be developed. We examined and optimized several key factors that affect genetic transformation of *J. curcas* in this study. The results showed that the EHA105 strain was superior to the other three *Agrobacterium tumefaciens* strains for infecting *J. curcas* cotyledons, and the supplementation of 100 mM acetosyringone slightly increased the transient transformation frequency. Use of the appropriate inoculation method, optimal kanamycin concentration and appropriate duration of delayed selection also improved the efficiency of stable genetic transformation of *J. curcas*. The percentage of β-glucuronidase positive *J. curcas* shoots reached as high as 56.0 %, and 1.70 transformants per explant were obtained with this protocol. Furthermore, we optimized the root-inducing medium to achieve a rooting rate of 84.9 %. Stable integration of the T-DNA into the genomes of putative transgenic lines was confirmed by PCR and Southern blot analysis. Using this improved protocol, a large number of transgenic *J. curcas* plantlets can be routinely obtained within approximately 4 months. The detailed information provided here for each step of *J. curcas* transformation should enable successful implementation of this transgenic technology in other laboratories.

**Electronic supplementary material:**

The online version of this article (doi:10.1007/s11816-015-0377-0) contains supplementary material, which is available to authorized users.

## Introduction

With the decreasing supplies of fossil fuels and the worsening of environmental pollution, biodiesel has received increasing attention as an alternative fuel. *Jatropha curcas* is considered a potential oilseed crop for biofuel production because its seeds contain up to 40 % oil, which can be easily converted to biodiesel or bio-jet fuel and used to partially or fully replace fossil fuel (Fairless 2007; Juan et al. 2011; Makkar and Becker 2009). In addition, *J. curcas* can grow under climate and soil conditions that are unsuitable for food production (Maghuly and Laimer 2013), and it is a renewable feedstock for producing soap, green fertilizers, pesticides and medicines (Kumar and Sharma 2008). However, at present, *J. curcas* does not contribute to the biofuel industry because the yield of its seeds is generally low in many areas, and the investment in large-scale cultivation of *J. curcas* had moved ahead of the scientific studies aiming to fully explore this plant and understand its limitations (Sanderson 2009).

Breeding of varieties that produce high and stable yields is one of the most efficient approaches to making *J. curcas* into a successful biofuel crop. Because of the low genetic diversity of *J. curcas* (Rosado et al. 2010; Sun et al. 2008), conventional breeding technology has limited potential for use in the genetic improvement of this plant (Sujatha et al. 2008). Transgenic breeding techniques can complement conventional breeding technology and have many advantages, such as directional cultivation of new breeds, reduced costs, a shorter breeding period, and the ability to introduce genes for traits that may not be available within the species or may be difficult to introduce via conventional breeding methods (Herr and Carlson 2013; Li et al. 1996; Visarada et al. 2009). Moreover, with the completion of genomic sequencing analysis (Hirakawa et al. 2012; Sato et al. 2011), expressed sequence tag analysis (Chen et al. 2011; Eswaran et al. 2012; Natarajan et al. 2010), and transcriptomic studies (Costa et al. 2010; Natarajan and Parani 2011; Zhang et al. 2014), *J. curcas* is poised to become a new model woody plant. Genetic transformation may become an important method for molecular breeding and gene function analysis of *J. curcas*.

The efficiency of the in vitro plant regeneration system, the efficiency of *Agrobacterium tumefaciens* transformation, and the specific antibiotic selection procedure are key factors in plant genetic transformation (Kajikawa et al. 2012; Li et al. 2008; Mao et al. 2009). To date, shoot regeneration systems for *J. curcas* have been successfully established using various explants such as cotyledons, epicotyls, hypocotyls, leaves, petioles, nodes, and stems (Khurana-Kaul et al. 2010; Kumar and Reddy 2010; Sharma et al. 2011; Singh et al. 2010; Sujatha and Mukta 1996; Toppo et al. 2012). Some regeneration systems have been utilized in genetic transformation protocols employing *A. tumefaciens* (Khemkladngoen et al. 2011; Kumar et al. 2010; Li et al. 2008; Mao et al. 2009; Misra et al. 2012; Pan et al. 2010) or particle bombardment (Joshi et al. 2011; Purkayastha et al. 2010). Due to several advantages of *Agrobacterium*-mediated genetic transformation, including the defined integration of transgenes, potentially low copy number, and preferential integration into transcriptionally active regions of the chromosome (Newell 2000), this method is the most widely used to generate transgenic *J. curcas*. Several *A. tumefaciens* strains have been used in the genetic transformation of *J. curcas*, including LBA4404 (Kumar et al. 2010; Li et al. 2008; Misra et al. 2012), EHA105 (He et al. 2009; Jaganath et al. 2014; Mazumdar et al. 2010), GV3101 (Qin et al. 2014), and AGL1 (Mao et al. 2009). The types of selectable markers and the selection pressure also play important roles in the genetic transformation of different explants. Using the herbicide phosphinothricin as a selective agent, Li et al. (2008) first reported the generation of transgenic *J. curcas*. Thereafter, several groups found that transgenic *J. curcas* can be regenerated by selection using hygromycin (Joshi et al. 2011; Kumar et al. 2010; Mao et al. 2009), kanamycin (Khemkladngoen et al. 2011; Misra et al. 2012; Pan et al. 2010), or bispyribac-sodium salt (Kajikawa et al. 2012). Many protocols required multiple handling steps and frequent changes of different medium, which not only require additional time but also may introduce contamination. The regeneration of transformed shoots takes 4–7 months, and the transformation efficiency is 4–53 % (Kajikawa et al. 2012; Khemkladngoen et al. 2011; Kumar et al. 2010; Li et al. 2008; Misra et al. 2012). Therefore, a simple, highly efficient, and rapid *Agrobacterium*-mediated genetic transformation system for *J. curcas* should be developed. Notably, root induction of regenerated shoots is also an important step for obtaining transgenic plants. Several root-inducing mediums (RIM) have been successfully used in the root induction of regenerated *J. curcas* shoots, but the rooting efficiency varied greatly, from 16 to 86 % in 1/2 MS medium supplemented with 0.2–0.5 mg L^−1^ IBA (Kajikawa et al. 2012; Khemkladngoen et al. 2011; Li et al. 2008; Toppo et al. 2012). Mazumdar et al. (2010) reported that the rooting induction efficiency of the *J. curcas* shoots was 75 % in 1/2 MS containing 5.0 μM (0.9 mg L^−1^) NAA within 17–20 days of cultivation. So RIM should also be optimized to ensure high and stable rooting efficiency of transformed shoots.

We have previously developed a simple and efficient *Agrobacterium*-mediated genetic transformation system for *J. curcas* using the cotyledons from mature seeds as explants (Pan et al. 2010). In this simple protocol, all stages of cultivation, including co-cultivation, callus initiation and proliferation, selection of transgenic calli, and regeneration of shoots, were performed on a single medium (MS-Jc1: MS medium supplemented with 3 mg L^−1^ BA and 0.01 mg L^−1^ IBA) with different antibiotics (Pan et al. 2010). However, the transformation efficiency of this method is low and variable (Kajikawa et al. 2012); thus, it requires substantial improvement.

In the present study, we further improved the protocol described previously by Pan et al. (2010) by optimizing several key factors including the supplementation of acetosyringone (AS), selection of *A. tumefaciens* strains, scheme of transformant selection, determination of antibiotic selection pressure, and formulation of RIM. This improved protocol substantially increases the regeneration efficiency of transgenic *J. curcas*; the percentage of β-glucuronidase (GUS)-positive shoots reached an average of 56.0, and 84.9 % of the transgenic shoots successfully rooted. Moreover, this protocol includes additional details that were not previously described by Pan et al. (2010).

## Materials and methods

### Preparation of explants

Mature *J. curcas* L. seeds were collected from trees grown in Xishuangbanna Tropical Botanical Garden (21°54′N, 101°46′E, 580 m asl) at the Chinese Academy of Sciences, located in Mengla County, Yunnan Province, China. The husked seeds were soaked in sterile water for 10–12 h at room temperature. The seeds were then surface sterilized with 75 % (v/v) ethanol for 1 min, followed by soaking for 15 min in 1.1 % (w/v) sodium hypochlorite and 0.1 % (v/v) Tween-20 with occasional agitation. Subsequently, the seeds were rinsed three times with sterile distilled water. The sterilized seeds were carefully dissected with a scalpel, and the embryos were removed from the seeds. Approximately 3/4 of the papery cotyledons were excised by cutting off the base of the cotyledons and the embryo axes, as shown in Pan et al. (2010). These cotyledons were used as explants for *Agrobacterium*-mediated genetic transformation.

### *Agrobacterium tumefaciens* strains and vector used for transformation

The *A. tumefaciens* strains LBA4404, EHA105, GV3101, and AGL1, all of which harbor the binary vector pCAMBIA2301 (CAMBIA, Canberra, Australia), were used for transformation experiments. EHA105 carrying *35S:AtFT* was used to evaluate the transformation method in subsequent transformation experiments. A single colony of *A. tumefaciens* was inoculated into 5 mL of liquid yeast extract and beef (YEB) medium containing 50 mg L^−1^ kanamycin and 25 mg L^−1^ streptomycin or 50 mg L^−1^ rifampicin and grown overnight at 28 °C on a rotary shaker at 200 rpm. An aliquot (0.5 mL) of the overnight culture was inoculated into 50 mL of liquid YEB medium containing the same antibiotics and allowed to grow at 28 °C with vigorous shaking until the OD_600_ reached approximately 0.6–1.0. *A. tumefaciens* cells were then collected by centrifugation at 5000 rpm for 5 min at 4 °C and re-suspended at OD_600_ = 0.4 with liquid Murashige and Skoog (MS) medium (Murashige and Skoog 1962) supplemented with 100 μM AS. The bacterial suspension was allowed to stand for approximately an hour before use.

### Transformation, selection, and regeneration of transformants

The excised cotyledon explants were inoculated with the *A. tumefaciens* suspension by co-incubation with shaking on a rotary shaker for 20 min at 150 rpm. The temperature was maintained at 28 °C. The explants were then transferred onto sterile filter paper to remove excess *A. tumefaciens* before being cultured on co-cultivation medium (CCM: MS-Jc1 supplemented with 100 μM AS) for 3 days at 26 ± 2 °C in the dark. Following co-cultivation, the explants were washed three times with sterile water containing 500 mg L^−1^ cefotaxime to eliminate *A. tumefaciens* and blotted dry on sterile filter paper. Then, they were transferred initially to callus-inducing medium [CIM: MS-Jc1 supplemented with 300 mg L^−1^ cefotaxime and 100 mg L^−1^ ticarcillin and clavulanate potassium (timentin)] for callus initiation and growth and subsequently cultured for 1, 2, or 3 weeks (Table 1). The explants were cultured under a 14 h light/10 h dark cycle with fluorescent light (100 μ Em^−2^ s^−1^) at 26 ± 2 °C. Then, the swollen cotyledon explants with small calli were placed on shoot-inducing medium (SIM: MS-Jc1 supplemented with 300 mg L^−1^ cefotaxime, 100 mg L^−1^ timentin, and 20, 30, or 40 mg L^−1^ kanamycin) with 1 cm of the cut end of each cotyledon inserted into the medium. After the 3-week culture period, the explants were individually subcultured to fresh SIM. Resistant calli and shoots were subcultured at 3-week intervals. The number of shoots per explant, frequency of positive GUS staining and transgene incorporation were scored after 12 weeks of cultivation on SIM.

**Table 1:** Effects of kanamycin concentration and duration of delayed selection on the regeneration and transformation efficiency of *J. curcas*

Kanamycin concentration (mg L^−1^)	Duration of delayed selection (weeks)	Number of explants*	Number of resistant shoots/explant**	Frequency of GUS-positive shoots (%)***	Number of GUS-positive shoots/explant
20	1	120	5.56 ± 0.33^Aa^	12.7 ± 3.1^Ded^	0.70 ± 0.15^Cc^
20	2	116	4.06 ± 0.35^Bb^	5.3 ± 1.2^Ee^	0.21 ± 0.04^Dde^
20	3	100	3.46 ± 0.26^BCbc^	1.3 ± 1.2^Ee^	0.05 ± 0.04^De^
30	1	111	3.35 ± 0.33^BCbc^	36.0 ± 2.0^Bb^	1.19 ± 0.18^Bb^
30	2	112	2.67 ± 0.29^CDcd^	27.3 ± 2.3^Cc^	0.70 ± 0.08^CDcd^
30	3	109	2.09 ± 0.18^CDd^	14.0 ± 2.0^Dd^	0.29 ± 0.03^Dde^
40	1	98	3.03 ± 0.22^Cc^	56.0 ± 3.5^Aa^	1.70 ± 0.15^Aa^
40	2	110	2.39 ± 0.21^CDcd^	33.3 ± 2.3^BCbc^	0.80 ± 0.03^BCc^
40	3	110	1.88 ± 0.17^Dd^	20.7 ± 3.1^CDcd^	0.39 ± 0.05^CDd^

* Each value is the mean ± SE of three independent experiments, each with 30–40 cotyledons. The cotyledons that were not green or that were contaminated by *A. tumefaciens* were not counted. ** Shoots of at least 1.5 cm in length were counted. ***Percentage of GUS-positive shoots calculated as the number of GUS-positive shoots/total number of resistant shoots × 100. Values with different lowercase letters are significantly different (*P* < 0.05, Tukey’s test). Values with different uppercase letters are significantly different (*P* < 0.01, Tukey’s test)

### Rooting and acclimation

Green and healthy regenerated shoots with three to four leaves were excised and transferred to root-inducing medium (RIM: half-strength (1/2) MS medium supplemented with 100 mg L^−1^ timentin, 1.0 % sucrose, 0.25 % carrageenan, and different combinations of IBA and NAA) (Table 2). The percentage of root induction was recorded and evaluated after 4 weeks and was calculated by comparing the number of rooting shoots to the total number of shoots. Rooted plantlets were carefully removed from the medium, washed thoroughly in running water to remove RIM attached to the roots, soaked in 0.1 % w/v carbendazim for 2 h, and then planted in polythene cups filled with sterilized soil consisting of humus:peat:vermiculite (3:1:1), covered with transparent plastic film to maintain humidity, and grown in the greenhouse at 22 ± 2 °C under a 16/8 h (light/dark) photoperiod with fluorescent light (100 μ Em^−2^ s^−1^). After 2 weeks, the transparent plastic film was removed, and the plants were sprayed with water to avoid wilting of the leaves in the first 2 days. The number of surviving plants was recorded after an additional 1–2 weeks.

**Table 2:** Effects of different concentrations of IBA and NAA in root-inducing medium (RIM) on the induction of roots from *J. curcas* shoots

IBA (mg L^−1^)	NAA (mg L^−1^)	Number of tested shoots*	Efficiency of root induction (%)**
0	0	86	5.8 ± 1.3^De^
0	0.1	85	32.8 ± 3.9^Cd^
0	0.2	88	65.0 ± 3.5^Bb^
0.1	0	86	9.2 ± 2.7^De^
0.1	0.1	87	58.5 ± 3.4^BCbc^
0.1	0.2	87	48.2 ± 2.6^Cc^
0.2	0	90	13.3 ± 2.3^De^
0.2	0.1	85	84.9 ± 3.7^Aa^
0.2	0.2	87	41.3 ± 3.2^Ccd^

* Each value is the mean ± SE of two independent experiments, each with 40–50 shoots. IBA, indole-3-butyric acid; NAA, α-naphthaleneacetic acid. ** Data were acquired after 4 weeks. Percentage root induction was calculated as the number of rooting shoots/total number of shoots × 100. Values with different lowercase letters are significantly different (*P* < 0.05, Tukey’s test). Values with different uppercase letters are significantly different (*P* < 0.01, Tukey’s test)

### Molecular analysis of transgenic plantlets

Genomic DNA was isolated from the leaves of putative transgenic plants and controls using the cetyl trimethylammonium bromide method (Allen et al. 2006). PCR analysis of the isolated genomic DNA was performed to identify transgenes in putative transformants using primers (Table S1) for the *GUS* and *NPTII* genes. A *35S CaMV* promoter primer and a *GUS*-specific primer were used to detect the *GUS* gene, which produced a 778-bp fragment. The *NPTII* gene was amplified with the *NPTII*-specific primer pair, producing a 679-bp fragment. PCR amplification reactions were performed in a 20-μL volume, and the PCR parameters were as follows: 94 °C for 3 min, followed by 30 cycles of 94 °C for 30 s, 61 °C (for *GUS*)/58 °C (for *NPTII*) for 30 s and 72 °C for 1 min with a final extension of 10 min at 72 °C. The plasmid pCAMBIA2301 was used as a positive control, and non-transgenic plant DNA was used as a negative control. PCR amplification products were separated on 1.0 % (w/v) agarose gels and visualized by ethidium bromide staining under UV light.

For Southern blot analysis, 5 µg of genomic DNA from the PCR-positive leaves was digested with *Eco*RI and *Xho*I (*Eco*RI and *Xho*I cut the pCAMBIA2301 plasmid once outside of the *GUS* gene) overnight at 37 °C to cleave a unique site in the T-DNA. Following digestion, the DNA fragments were separated on 0.8 % (w/v) agarose gels, which were then processed and transferred to Hybond-N^+^ membranes (Roche Diagnostics, Mannheim, Germany) following the standard procedure (Sambrook et al. 2001). The 483-bp fragment of the *GUS* probe was labeled with digoxigenin (DIG) using a Roche PCR DIG Probe Synthesis Kit. Hybridization and chemiluminescent detection of the blots were performed following the manufacturer’s instructions (Roche). Total RNA of *35S:AtFT* transgenic lines and controls was extracted from frozen tissue as described by Ding et al. (Ding et al. 2008). First-strand cDNA was synthesized using the PrimeScript^®^ RT Reagent Kit with gDNA Eraser (TAKARA, Dalian, China) according to the manufacturer’s instructions. The primers used for RT-PCR are listed in Table S1.

### Histochemical staining of β-glucuronidase (GUS) activity

Histochemical staining of GUS activity was performed according to the method of Jefferson et al. (1987) with some modifications. Transient *GUS* expression in cotyledon explants was monitored at 3 days after *A. tumefaciens* transformation, and stable *GUS* expression was examined in kanamycin-resistant calli, leaf lamina of positive shoots, stems, roots, flowers, young fruits of putative T_0_ transgenic adult plants, T_1_ cotyledons from GUS-positive plants, and the same tissues of the control plants. These samples were immersed in GUS staining solution (50 mM sodium phosphate (pH 7.0), 0.5 mM K_3_Fe(CN)_6_, 0.5 mM K_4_Fe(CN)_6_·3H_2_O, 0.5 % Triton X-100 and 1 mM X-gluc) and subjected to vacuum for 20 min. After incubation in the GUS staining solution at 37 °C overnight, the samples were destained in 70 % ethanol for several hours to visualize the GUS-stained areas. The efficiency of transient transformation was calculated by comparing the number of GUS-stained cotyledons to the total number of cotyledons infected by *A. tumefaciens*. The percentage of GUS-positive shoots was calculated by comparing the number of GUS-stained leaves to the total number of leaves from kanamycin-resistant shoots.

### Statistical analysis

In this study, 30–50 explants were used for each experimental condition, and each experiment was repeated three times. The efficiency of transient transformation, the percentage of GUS-positive shoots and root induction were analyzed using the Statistical Product and Service Solution software (version 16.0; SPSS Inc., Chicago, IL, USA), and the data are presented as the mean ± standard error of the repeated experiments. Analysis of variance among the means was determined using one-way ANOVA with Tukey’s post hoc test.

## Results

### Influence of AS and *A. tumefaciens* strains on the efficiency of transient transformation

**Fig. 1:**
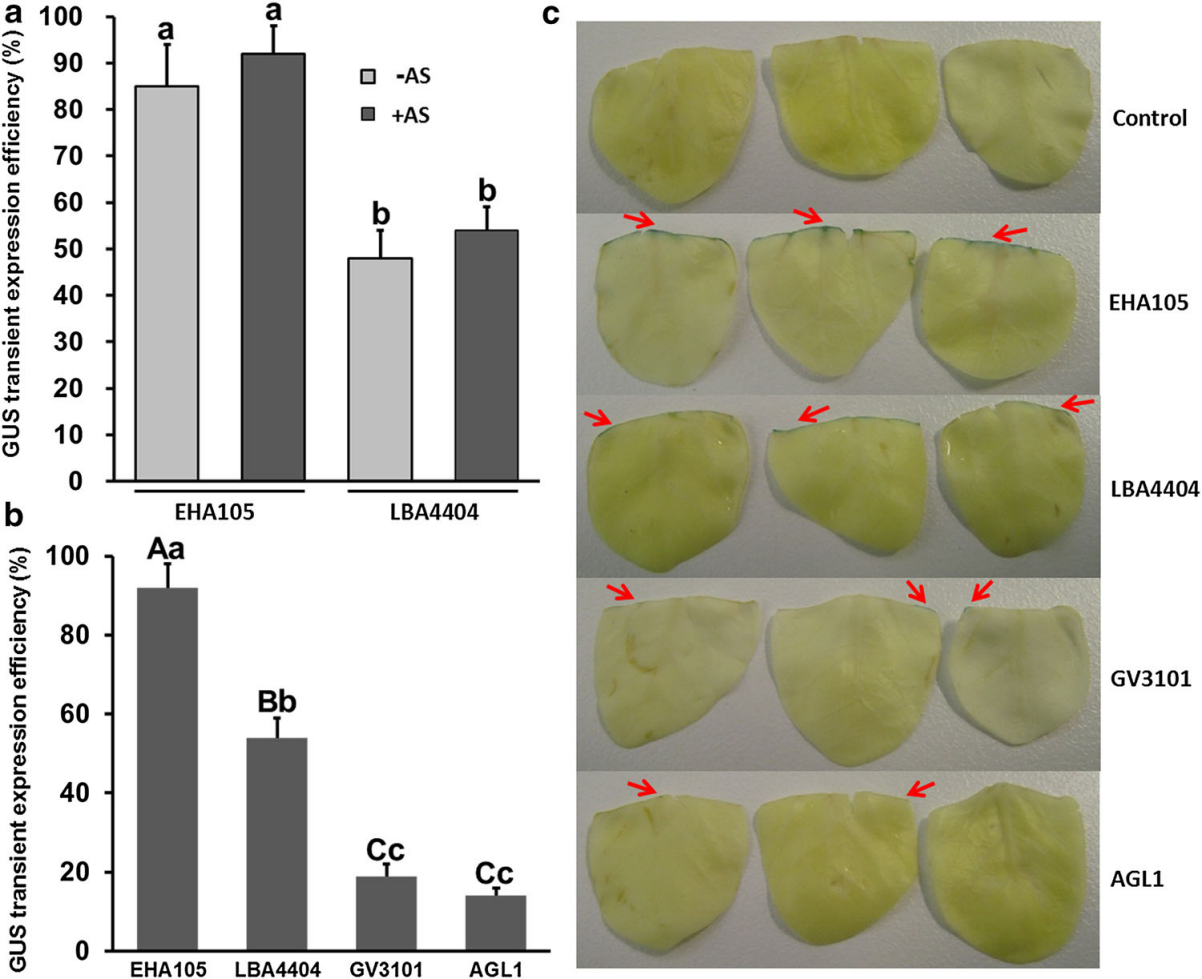
Efficiency of transient transformation of *J. curcas* cotyledon explants after 3 days of co-cultivation with *Agrobacterium tumefaciens*. **a** Influence of AS on the efficiency of transient transformation. **b** Influence of *Agrobacterium* strains on the efficiency of transient transformation. **c** Transient GUS expression differed in the incisions of cotyledons after co-cultivation with different *Agrobacterium* strains. Each value is the mean ± SE of three independent experiments, each including 30 cotyledons. Values with different *lowercase letters* are significantly different (*P* < 0.05, Tukey’s test). Values with different *uppercase letters* are significantly different (*P* < 0.01, Tukey’s test). *Red arrows* indicate GUS staining on cotyledon explants

To determine the effects of AS on the transformation efficiency of cotyledon explants of *J. curcas* infected with *A. tumefaciens*, the efficiency of transient transformation was analyzed by examining the GUS-stained cotyledons after 3 days of co-cultivation with *A. tumefaciens*. The addition of 100 mM AS during infection and co-cultivation slightly increased the transient transformation efficiency from 84 to 92 % using EHA105, and from 48 to 54 % using LBA4404, respectively (Fig. 1a). Therefore, 100 mM AS was used in the subsequent infection and co-cultivation experiments. To select the best *A. tumefaciens* strain for *J. curcas* transgenic studies, the transformation efficiencies of four *A. tumefaciens* strains harboring pCAMBIA2301—EHA105, LBA4404, GV3101, and AGL1—were examined. As shown in Fig. 1b, different *A. tumefaciens* strains exhibited significant differences in their ability to transform cotyledon explants of *J. curcas*. Compared with the other *A. tumefaciens* strains, EHA105 showed the highest transient transformation efficiency (92 %). The transient transformation efficiencies of LBA4404, GV3101, and AGL1 were approximately 54, 19 and 14 %, respectively (Fig. 1b). An intense blue color was observed in the incisions in cotyledons (Fig. 1c). The number of stained areas per EHA105-infected explant was significantly higher than that observed with other *A. tumefaciens* strains, whereas no GUS staining was detected in control explants transformed with EHA105 without the binary vector (Fig. 1c). Therefore, EHA105 was superior to the other three *A. tumefaciens* strains for infecting *J. curcas* cotyledons, and was selected for subsequent transformation experiments in this study.

### Optimization of kanamycin concentration and duration of delayed selection

**Fig. 2:**
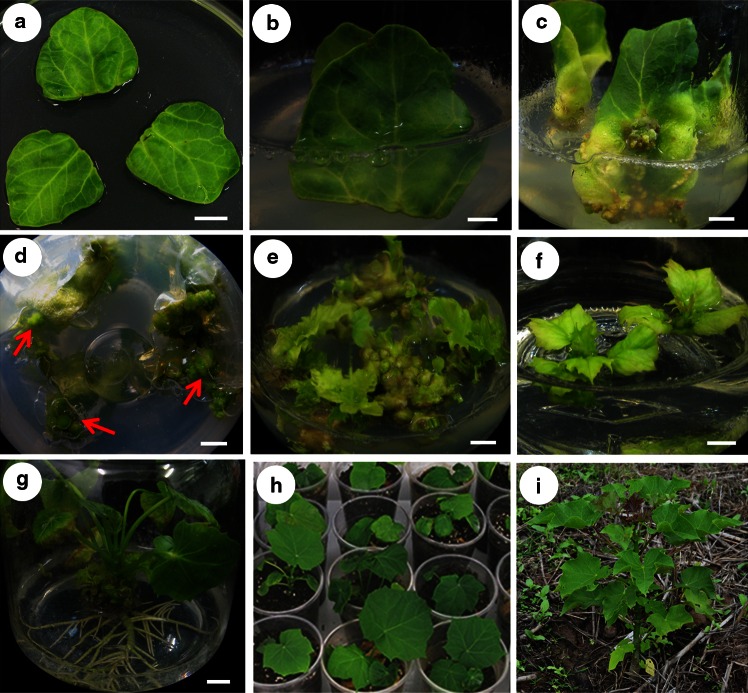
Different stages of *Agrobacterium*-mediated genetic transformation of *J. curcas*. **a** Swollen cotyledon explants incubated on CIM for 1 week. **b** Inserting cotyledon explants into SIM after cultivation on CIM for 7 days to induce resistant calli. **c** Three-week-old resistant calli in SIM under the condition of 1-week delayed selection with 40 mg L^−1^ kanamycin. **d** Resistant calli (indicated by *red arrows*) in SIM. **e** Resistant shoot buds on SIM under the condition of 1-week delayed selection with 40 mg L^−1^ kanamycin in the third cycle of selection. **f** Resistant plantlets were excised and transferred to RIM. **g** Putative transgenic plantlets rooted after incubation on RIM for 4 weeks. **h** Transgenic plants in pots acclimatized in the greenhouse for 4 weeks. **i** Growth of transgenic plantlets in the field. *Scale bars* represent 5 mm

As shown in our previous report of *J. curcas* transformation, cotyledon explants excised from mature seeds of *J. curcas* were very sensitive to the selection agent kanamycin, and 5 mg L^−1^ kanamycin in the medium completely inhibited callus formation from cotyledon explants (Pan et al. 2010). The regeneration frequency and positive transformation rate were relatively low when cotyledon explants were cultivated on CIM without kanamycin for 4 weeks after co-cultivation with *Agrobacterium* (Pan et al. 2010). To improve the efficiency of kanamycin selection in *J. curcas*, nine independent selection experiments were carried out with different kanamycin concentrations and duration of delayed selection (Table 1). After co-cultivation with *A. tumefaciens*, the cotyledon explants were then transferred onto CIM and cultured for 1, 2, or 3 weeks without selection. The cotyledon explants began to turn green and grow larger after 1 week (Fig. 2a), and some small calli were produced on the cut ends of the swollen cotyledon explants. The cotyledon explants were inserted into the SIM with the cut ends embedded in the medium (Fig. 2b). A clear difference was observed between the transformed and untransformed cotyledon explants during the kanamycin selection process: there were many green calli formed at the incisions of the transformed cotyledon explants (Additional file 1: Fig. S1 a and c), whereas no such resistant calli were formed from the untransformed cotyledon explants (Additional file 1: Fig. S1 b and d). The base of cotyledon explants embedded in the medium turned yellow, and they gradually formed many resistant calli at the incisions after 3 weeks under the condition of 1-week delayed selection with 40 mg L^−1^ kanamycin, whereas the upper section of cotyledon explants (above the medium) stayed green and continued to grow (Fig. 2c, d). Then, the resistant calli were excised and transferred onto new SIM for the second cycle of selection; during this period, some adventitious buds were found in the regions of the resistant calli. Resistant calli and shoots were subcultured at 3-week intervals. After three passages of selection, many resistant shoots were regenerated, as shown in Fig. 2e. Additional adventitious shoots were obtained during the fourth selection cycle. Resistant shoots that were 1.5–2 cm in length with 3–4 leaves were excised and cultured on RIM for inducing roots (Fig. 2f).

We utilized GUS staining as an indicator of successful transformation, and the *GUS* expression rate was calculated as follows: GUS-positive shoots/total kanamycin-resistant shoots (Table 1). The number of shoots per explant was measured to calculate the regeneration frequency. The regenerated shoots from the third and fourth cycles of kanamycin selection were statistically analyzed, and the leaves of these regenerated shoots were examined by GUS staining. As shown in Table 1, the regeneration frequency of shoots decreased significantly as the kanamycin concentration and the duration of delayed selection after co-cultivation increased. The highest regeneration frequency (5.56 shoots per explant) was observed under the condition of 1-week delayed selection with 20 mg L^−1^ kanamycin, whereas the lowest regeneration frequency (1.88 shoots per explant) was observed under the condition of 3-week delayed selection with 40 mg L^−1^ kanamycin (Table 1). More importantly, significant differences in the number of GUS-positive shoots per explant and the frequency of GUS-positive shoots were observed among different kanamycin concentrations and duration of delayed selection. The number of GUS-positive shoots per explant and the frequency of GUS-positive shoots were only 0.05 and 1.3 %, respectively, under the condition of a 3-week delayed selection with 20 mg L^−1^ kanamycin. When the kanamycin concentration was increased and the duration of delayed selection was reduced, the number of GUS-positive shoots per explant and the frequency of GUS-positive shoots increased gradually. Under the condition of a 1-week delayed selection with 40 mg L^−1^ kanamycin, the number of GUS-positive shoots per explant and the frequency of GUS-positive shoots reached maxima of 1.70 and 56.0 %, respectively (Table 1).

### Optimization of the root-inducing medium

To obtain high rooting efficiency, we optimized RIM used for root induction from regenerated shoots by adding different concentrations of IBA and NAA to 1/2 MS. As shown in Table 2, the rooting frequency increased with increases in the concentrations of IBA or NAA. The lowest rooting frequency was observed on 1/2 MS basal medium, only 5.8 %. A rooting frequency of approximately 65.0 % was observed on RIM with 0.2 mg L^−1^ NAA. The combination of 0.2 mg L^−1^ IBA and 0.1 mg L^−1^ NAA in RIM further increased the rooting percentage to its highest value (84.9 %) (Table 2). Adventitious roots were initiated on regenerated shoots incubated on RIM within 2 weeks, and the roots were much more numerous and stronger after incubation on RIM for 4 weeks (Fig. 2g). Rooted plantlets were planted in sterilized soil consisting of humus:peat:vermiculite (3:1:1) and acclimatized in the greenhouse at 22 ± 2 °C. After 4–5 weeks, more than 85 % of the acclimatized plantlets survived, and there was no obvious phenotypic difference among GUS transgenic *J. curcas* plants (Fig. 2h). Then, the plantlets were transferred to the fields for further growth (Fig. 2i).

### Confirmation of transgenic plants

**Fig. 3:**
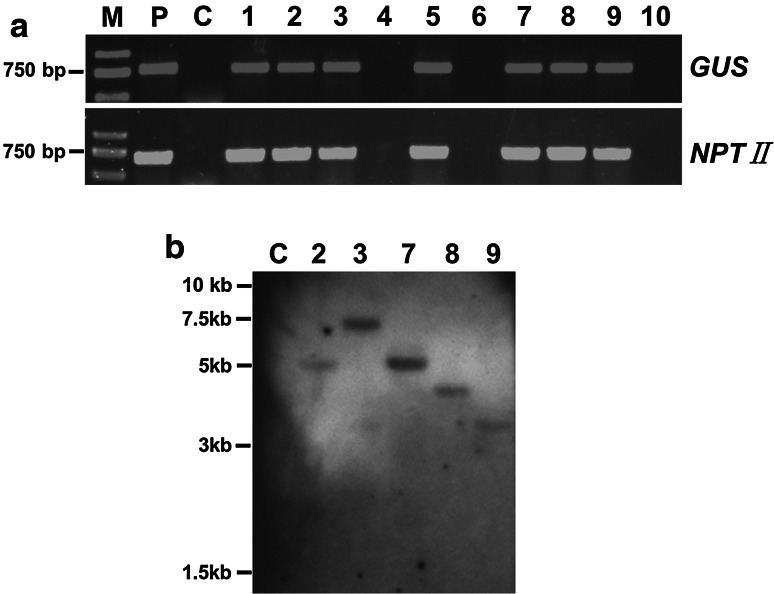
PCR and Southern blot analysis of transgenic *J. curcas* plants. **a** Amplification of the 778-bp *GUS* gene (*upper panel*) and the 679-bp *NPTII* gene (*lower panel*) in transgenic plants. Lanes: M, molecular size marker (Trans2 K DNA ladder); P, positive control (pCAMBIA2301 plasmid); C, negative control (non-transformed plant); 1–10, putative transgenic *J. curcas* lines. **b** Southern hybridization of genomic DNA isolated from negative control (a non-transformed plant) and PCR-positive shoots. Genomic DNA was digested with *Eco*RI and hybridized with a DIG-labeled *GUS* probe. Lanes: C, negative control (a non-transformed plant); 2, 3, 7, 8, and 9, five independent transgenic plants

To confirm the presence of the transgenes in the putative transgenic plants, genomic DNA was extracted from the leaves of non-transformed and putative transgenic plants in pots, which were selected with 40 mg L^−1^ kanamycin after cultivation on CIM for 1 week. Then, the reporter gene *GUS* and the selective marker gene *NPTII* were identified by PCR amplification. A 778-bp fragment corresponding to the *GUS* gene was detected in most of the putative transgenic plants (Fig. 3a, upper panel, lanes 1 – 10) and the positive control (Fig. 3a, upper panel, lane P), but no amplification product was found in the non-transformed control plant (Fig. 3a, upper panel, lane C). The expected 679-bp fragment of the *NPTII* gene was also detected in most of the putative transgenic plants (Fig. 3a, lower panel, lanes 1–10), whereas the control plant showed no amplification products (Fig. 3a, lower panel, lane C).

The integration of the *GUS* gene into the genome of transgenic *J. curcas* plants was further verified by Southern blot analysis of genomic DNA from PCR-positive transgenic plants. As shown in Fig. 3b, all of the tested plants showed one band after hybridization (Fig. 3b, lanes 2, 3, 7, 8 and 9), and no hybridization band was detected when control plant DNA was used (Fig. 3b, lane C). This result indicates that a single copy of the *GUS* gene had integrated into the genomes of the tested transgenic plants.

### GUS histochemical analysis

**Fig. 4:**
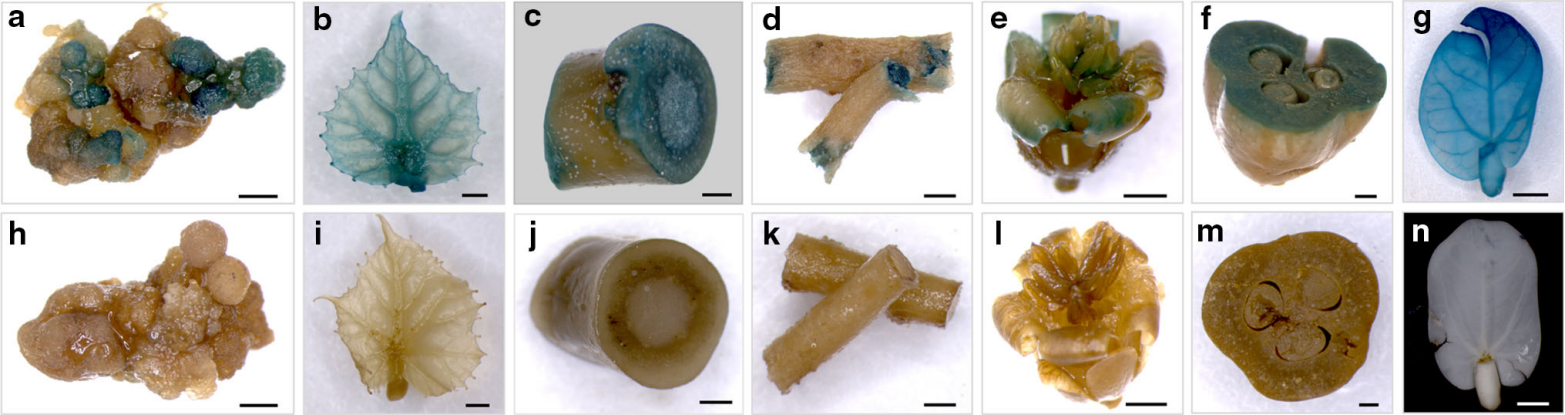
Histochemical GUS staining of various tissues of transgenic *J. curcas* Representative photos of GUS-stained calli (**a** and **h**), leaves (**b** and **i**), stems (**c** and **j**), roots (**d** and **k**), flowers (**e** and **l**), and fruits (**f** and **m**) from T_0_ transgenic plants (**a**-**f**) and control (**h**-**m**). Cotyledons from the progeny (T_1_) of transgenic (**g**) and control (**n**) plants are also shown. *Scale bars* represent 5 mm

The stable transformation events were further monitored using histochemical analysis of GUS activity. As shown in Fig. 4a and b, intense blue staining was visibly detected in a resistant callus and young leaf from a regenerated shoot under the condition of 1-week delayed selection with 40 mg L^−1^ kanamycin, whereas no such blue staining was detected in a non-transformed callus and leaf (control) (Fig. 4h, i). To visualize stable GUS expression in adult transgenic plants, we next analyzed the stems, roots, flowers, and fruits. Strong GUS staining was observed in the stems, roots, flowers and fruits of T_0_ adult transgenic plants (Fig. 4c–f), whereas no GUS expression was observed in the negative control plants (Fig. 4j–m). Positive GUS expression was also observed in cotyledons of the progeny (T_1_) of transgenic plants (Fig. 4g), whereas GUS activity was not detected in the cotyledons of the control plants (Fig. 4n), indicating that the transgene was stably inherited in T_1_ progeny.

### Overexpression of *Arabidopsis FLOWERING LOCUS T* in *J. curcas* causes early flowering in vitro

**Fig. 5:**
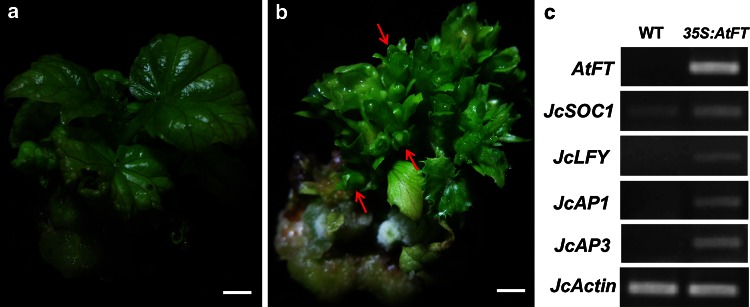
Phenotypes of WT and *35S:AtFT* transgenic *J. curcas* shoots cultured in vitro and semi-quantitative RT-PCR analysis of flowering genes in these lines. **a** WT *J. curcas* shoot cultured in vitro. **b** Flower buds of *35S:AtFT* transgenic *J. curcas* cultured in vitro. **c** Semi-quantitative RT-PCR analysis of flowering genes in the shoot apexes of WT and *35S:AtFT* transgenic *J. curcas* cultured in vitro. *Red arrows* indicate flower buds. *Scale bars* represent 1 cm

*FLOWERING LOCUS T* (*FT*) is a key gene mediating the vegetative-to-reproductive transition and is conserved in many flowering plants (Kardailsky et al. 1999; Li et al. 2014; Ye et al. 2014). Using the transformation protocol described above, we transferred *Arabidopsis FT* (*AtFT*) cDNA under the control of the CaMV 35S promoter into 30 cotyledon explants of *J. curcas*, and we obtained a total of 17 independent transgenic shoots in 2.5 months. Flower buds formed directly on most of the *35S:AtFT* transgenic shoots of *J. curcas* in vitro, whereas the control explants did not produce flower buds under the same conditions (Fig. 5a, b). Semi-quantitative reverse transcription PCR (RT-PCR) analysis of gene expression related to flower bud development in *J. curcas* was performed on the shoot apexes of the wild-type (WT) and *35S:AtFT* transgenic lines cultured in vitro. Consistent with the phenotype of the transgenic shoots, which showed early flowering in vitro, the transcript levels of *AtFT, JcSOC1*, *JcLFY*, *JcAP1*and *JcAP3* were significantly upregulated in the transgenic lines (Fig. 5c). These results demonstrate that the improved transformation protocol provides a reliable method for the introduction of functional genes into *J. curcas*.

## Discussion

As a potent inducer of *A. tumefaciens* for infecting plant cells, AS has been successfully employed to improve transformation efficiency in many plants (Bull et al. 2009; Ramamoorthy and Kumar 2012; Wang et al. 2012). Similar to the results obtained by Li et al. (2008) and Kumar et al. (2010), the addition of 100 mM AS in this study slightly enhanced GUS transient expression efficiency in *J. curcas* compared with the control (Fig. 1a). The *A. tumefaciens* strain is one of the most important factors potentially affecting the efficiency of genetic transformation. Different *Agrobacterium* strains have been successfully used to transform *J. curcas*; however, the transformation efficiency varied greatly among the different transformation methods (Jaganath et al. 2014; Kajikawa et al. 2012; Li et al. 2008). We found that EHA105 was superior to other *Agrobacterium* strains for genetic transformation of *J. curcas* cotyledons (Fig. 1b, c). Our Southern blot analysis showed that the 5 transgenic *J. curcas* lines that we tested each had a single copy of the transgene (Fig. 3b), which is consistent with the report that *Agrobacterium* strain EHA105 appeared to be capable of generating single-copy transgenic tomato plants (Chetty et al. 2013).

The in vitro regeneration ability of the explants is crucial for establishing a successful *Agrobacterium*-mediated transformation system. Different explants of *J. curcas* have been reported to be used in transformation (Kajikawa et al. 2012; Khemkladngoen et al. 2011; Kumar et al. 2010; Li et al. 2008; Mao et al. 2009; Misra et al. 2012; Pan et al. 2010). In many plants, cotyledon explants are better suited to regeneration and transformation than other explants (Li et al. 2008, 2006; Murthy et al. 2003). Cotyledons excised from mature seeds of *J. curcas*, which are available in large quantities for transformation year-round, were found susceptible to *A. tumefaciens* infection (Li et al. 2014; Pan et al. 2010; Tao et al. 2015). In the current study, multiple resistant calli could be rapidly induced from the cut edges of cotyledon explants after 2–3 weeks of incubation on SIM (Fig. 2c), and transgenic shoots were regenerated from these resistant calli (Fig. 2e).

The selection strategy is also a key factor in improving transformation efficiency. Many plant species, including *J. curcas*, have been found to be hypersensitive to kanamycin, and this hypersensitivity is most likely the source of the reduced transformation efficiencies observed in previous studies (Kajikawa et al. 2012; Pan et al. 2010). Alternative selection strategies, including reduced kanamycin concentrations and delayed selection, have been successful for obtaining transgenic plants in kanamycin–hypersensitive species, including apple (Yao et al. 1995) and almond (Miguel and Oliveira 1999; Ramesh et al. 2006). In this study, with increasing kanamycin concentrations and decreasing durations of delayed selection, the number of GUS-positive shoots increased (Table 1). A stable transformation rate of approximately 56.0 % and 1.70 GUS-positive shoots per explant were obtained under the condition of 1-week delayed selection with 40 mg L^−1^ kanamycin (Table 1). The success of selection depends not only on kanamycin concentrations and delayed selection but also on improving the contact between explants and medium during inoculation (Bhatia et al. 2005). The cotyledon explants transferred from CIM were inserted into SIM with approximately 1 cm of their cut ends embedded in the medium (Fig. 2b). After 2–3 weeks, green calli and adventitious buds developed at the cut ends of cotyledon explants in the medium (Fig. 2c, d). This improved inoculation method might also result in a reduced number of escapees from kanamycin selection. To our knowledge, this is the first report describing the insertion of cut ends of explants into selection medium during *Agrobacterium*-mediated transformation of *J. curcas*.

We found that the rooting efficiency of regenerated *J. curcas* shoots varied greatly among different RIMs, which agrees with previous studies (Kajikawa et al. 2012; Khemkladngoen et al. 2011; Li et al. 2008; Mazumdar et al. 2010). The differences in rooting efficiency might depend not only on the concentrations of IBA and NAA in RIMs but also on the condition of shoots and/or the hormone levels in SIMs in these reports. Using different combinations of IBA and NAA, we optimized the conditions for root induction of the regenerated shoots. The highest rooting efficiency (84.9 %) was observed in 1/2 MS supplemented with 0.2 mg L^−1^ IBA and 0.1 mg L^−1^ NAA (Table 2). For the regenerated shoots that are difficult to root, in vivo grafting could be employed to obtain transgenic *J. curcas* plants (Jaganath et al. 2014; Li et al. 2014). In addition, transgenic *J. curcas* shoots overexpressing *AtFT* driven by the strong constitutive 35S promoter, which were obtained in this study and produced flower buds in vitro (Fig. 5b), were not able to develop into normal plants. As we showed previously with *J. curcas FT* homolog (Li et al. 2014), the use of weaker constitutive promoters, tissue specific promoters, or inducible promoters will be required to produce normal transgenic *J. curcas* plants overexpressing *AtFT*.

**Fig. 6:**
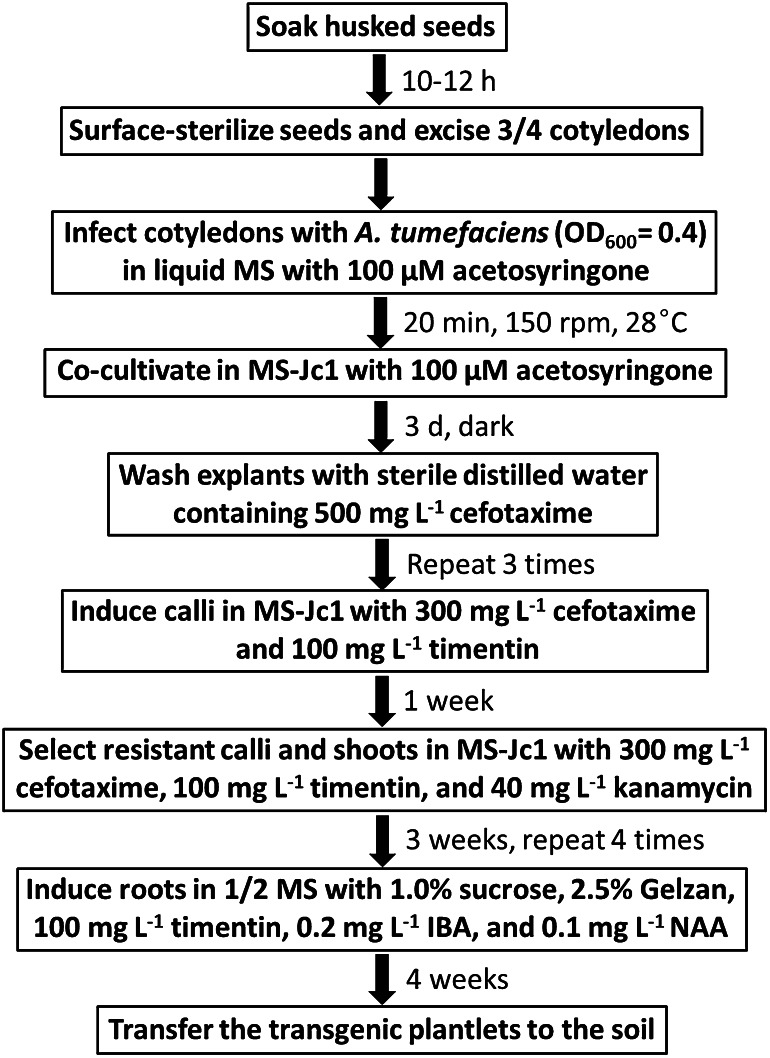
Schematic representation of the improved protocol for *Agrobacterium*-mediated genetic transformation and regeneration of *J. curcas* using cotyledons as explants

In conclusion, several important factors that affect the efficiency of *Agrobacterium*-mediated genetic transformation of *J. curcas* were optimized in this study, including the supplementation of AS, *A. tumefaciens* strain, kanamycin concentration, duration of delayed selection, inoculation method, and root-inducing medium, which have not been tested in our previous study (Pan et al. 2010). Compared to our previous protocol, genetic transformation and rooting efficiency of the improved protocol were greatly enhanced and reached an average of 56.0 and 84.9 %, respectively. The overall scheme is presented in Fig. 6. The protocol requires only two basal media and takes approximately 4 months from the start of co-cultivation to the rooting of plantlets. This improved transformation protocol is simple and reproducible; thus, it may be useful for studies of functional genes and genetic improvement of *J. curcas*. And the genetic transformation strategy reported here may be applied to other plants with various selective agents and explant types.

## Electronic supplementary material

Fig. S1. Development of resistant callus of *J. curcas* in selection medium. (a, c) Resistant callus (indicated by red arrows) developing from the incisions of transformed cotyledon explants in selection medium supplemented with 40 mg L^−1^ kanamycin for 2 weeks. (b, d) No resistant callus was detected among the untransformed cotyledon explants (control). (TIFF 1976 kb)

Table S1. Primers used in this study (DOCX 16 kb)
