# Supplementary figures and images for: An efficient protocol for Agrobacterium-mediated transformation of the biofuel plant Jatropha curcas by optimizing kanamycin concentration and duration of delayed selection
Source: Plant Biotechnol Rep. 2015 Nov 6;9(6):405–16. doi: 10.1007/s11816-015-0377-0 (PMC4662722; doi:10.1007/s11816-015-0377-0)

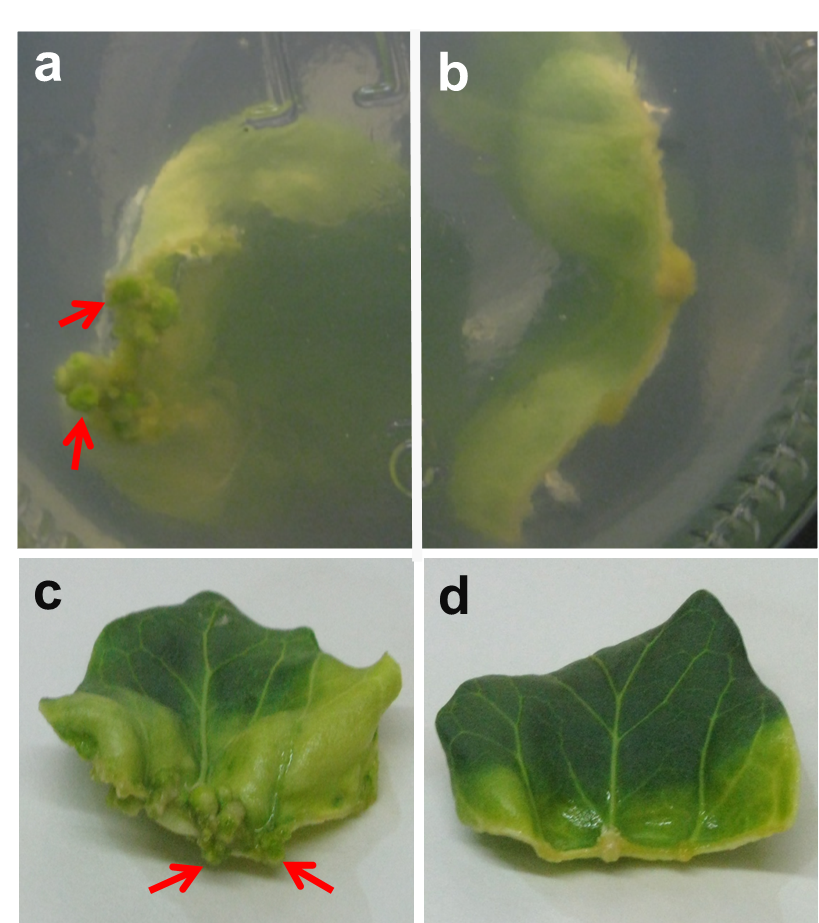

Supplement: Supplementary file 1 — Fig. S1. Development of resistant callus of J. curcas in selection medium. (a, c) Resistant callus (indicated by red arrows) developing from the incisions of transformed cotyledon explants in selection medium supplemented with 40 mg L−1 kanamycin for 2 weeks. (b, d) No resistant callus was detected among the untransformed cotyledon explants (control). (TIFF 1976 kb) [file 11816_2015_377_MOESM1_ESM.tif]
