# Supplementary material for: An efficient protocol for Agrobacterium-mediated transformation of the biofuel plant Jatropha curcas by optimizing kanamycin concentration and duration of delayed selection
Source: Plant Biotechnol Rep. 2015 Nov 6;9(6):405–16. doi: 10.1007/s11816-015-0377-0 (PMC4662722; doi:10.1007/s11816-015-0377-0)
Supplement: Supplementary file 2 — Table S1. Primers used in this study (DOCX 16 kb) [file 11816_2015_377_MOESM2_ESM.docx]

**Table S1. Primers used in this study**

| **Primer** | **Sequence (from 5’ to 3’)** |
| --- | --- |
|  |  |
| *35S*-F | GCTCCTACAAATGCCATCATTGCGATA |
| *GUS*-R | ATTGACCCACACTTTGCCGTAATGAG |
| *NPTII*-F | GGCGATACCGTAAAGCACGAGGAA |
| *NPTII*-R | CTATGACTGGGCACAACAGACAAT |
| *AtFT-*F | GAACAACCTTTGGCAATGAGA |
| *AtFT-*R | TCTTCCTCCGCAGCCACT |
| *JcSOC1*-F | CTCTCGGAAAAGTGTGGGATC |
| *JcSOC1*-R | TTCTTGGACGGCAACGCTTA |
| *JcLFY*-F | GGATAAGATACTACACAGCAGCGA |
| *JcLFY*-R | TAACCCTTCTTGAGAGAGAGCATC |
| *JcAP1*-F | GCTTCAACACTTAGAGCACCAG |
| *JcAP1*-R | GCTTAGCAAGTTGTTTTGGTCA |
| *JcAP3*-F | TCTCTTCGGTTTTGTAGTAGTGGGTTT |
| *JcAP3*-R | AGAACAGTGAGTTGCTTGAGCTTTTTT |
| *JcActin1*-F | CTCCTCTCAACCCCAAAGCCAA |
| *JcActin1*-R | CACCAGAATCCAGCACGATACCA |
